# Limited Effects of Set Shifting Training in Healthy Older Adults

**DOI:** 10.3389/fnagi.2017.00069

**Published:** 2017-03-23

**Authors:** Petra Grönholm-Nyman, Anna Soveri, Juha O. Rinne, Emilia Ek, Alexandra Nyholm, Anna Stigsdotter Neely, Matti Laine

**Affiliations:** ^1^Department of Psychology, Åbo Akademi UniversityTurku, Finland; ^2^Turku PET Centre, University of TurkuTurku, Finland; ^3^Student Health CarePorvoo, Finland; ^4^Department of Social and Psychological Studies, Karlstad UniversityKarlstad, Sweden; ^5^Turku Brain and Mind Center, University of TurkuTurku, Finland

**Keywords:** set shifting, task switching, cognitive training, executive functions, normal aging

## Abstract

Our ability to flexibly shift between tasks or task sets declines in older age. As this decline may have adverse effects on everyday life of elderly people, it is of interest to study whether set shifting ability can be trained, and if training effects generalize to other cognitive tasks. Here, we report a randomized controlled trial where healthy older adults trained set shifting with three different set shifting tasks. The training group (*n* = 17) performed adaptive set shifting training for 5 weeks with three training sessions a week (45 min/session), while the active control group (*n* = 16) played three different computer games for the same period. Both groups underwent extensive pre- and post-testing and a 1-year follow-up. Compared to the controls, the training group showed significant improvements on the trained tasks. Evidence for near transfer in the training group was very limited, as it was seen only on overall accuracy on an untrained computerized set shifting task. No far transfer to other cognitive functions was observed. One year later, the training group was still better on the trained tasks but the single near transfer effect had vanished. The results suggest that computerized set shifting training in the elderly shows long-lasting effects on the trained tasks but very little benefit in terms of generalization.

## Introduction

Executive functions represent higher-level cognitive control processes that are crucial for everyday activities. Different models of the mental architecture of executive functions have been put forth, but a particularly influential model by Miyake et al. (2000) that is based on data from young adults postulates three major executive functions that are separable but strongly interrelated. These functions are (1) working memory updating, (2) inhibition of task-irrelevant responses, and (3) shifting between tasks and mental sets. A later study gave support for the tripartite model of executive functions also in older adults (Vaughan and Giovanello, 2010). All three functions, including the third one that is at the focus of the present study, have been found to decline with older age (Cepeda et al., 2001; Kray et al., 2004; Zelazo et al., 2004). Little research interest has been directed to the trainability of set shifting in late adulthood, despite the fact that the ability to switch sets or tasks quickly is important in our everyday life (Monsell, 2003; Vaughan and Giovanello, 2010). Moreover, as the risk of cognitive impairment is enhanced in late adulthood due to, for example, dementing disorders, there is a need for finding suitable compensatory interventions for older adults. Therefore, we set out to study the effects of set shifting training in older adults with a 5-week adaptive training regime.

Although the generalizability of set shifting training in healthy elderly adults has been scarcely studied, there is an increasing number of studies on the effects of computerized working memory and multidomain training in healthy elderly (Buschkuehl et al., 2008; Dahlin et al., 2008b; Borella et al., 2010; Brehmer et al., 2011, 2012; Barnes et al., 2013; Zinke et al., 2013; Sandberg et al., 2014). In addition to improvement on the trained task itself, many of these recent training studies have shown that training can lead to *near transfer*, that is, improvement on tasks that are closely related to the intervention (e.g., working memory training leading to improved performance on another working memory task). Some findings suggest that training may even show *far transfer*, that is, generalization to other cognitive domains (e.g., working memory training leading to improved performance on a task measuring fluid intelligence). The results from a recent meta-analysis by Karbach and Verhaeghen (2014) indicated that training of working memory and executive functions was effective in older persons both with regard to near and far transfer, albeit the latter transfer effect was more modest. However, a re-analysis by Melby-Lervåg and Hulme (2016) found no convincing support for far transfer following working memory training in older age.

The results from the few existing set shifting training studies investigating transfer effects have varied, but most of them have found near transfer effects (Minear and Shah, 2008; Karbach and Kray, 2009; Pereg et al., 2013; Soveri et al., 2013). To our knowledge, only Karbach and Kray (2009) have included elderly adults in their set shifting training study. They found near transfer effects in reaction times to a set-shifting task structurally similar to the trained task for children, young adults, and older adults both with regard to switching cost and mixing cost when compared with the respective active control groups. *Switching cost* refers to mean reaction times (RTs) of switch trials minus mean RTs of non-switch trials within a mixed block, i.e., within the task block where switching takes place. *Mixing cost* refers to mean RTs of nonswitch trials in a mixed block minus mean RTs of single task trials where no switching takes place (see also the next paragraph for more information about switching and mixing cost). The effects were most pronounced in children and older participants on the mixing cost. Additionally, far transfer was found to inhibition, verbal and spatial working memory, and fluid intelligence in all age groups. The training tasks of Karbach and Kray (2009) were later used by Zinke et al. (2012) who studied transfer effects of set shifting training in adolescents. They found that compared to controls, set shifting training resulted in transfer to the mixing cost in a similar but untrained set shifting task, but far transfer was limited to a speed task and a tendency toward faster performance in an updating task. Thus, their transfer results were more limited than those of Karbach and Kray (2009). Also Pereg et al. (2013), studying set shifting training in young adults, used the same paradigm as Karbach and Kray (2009) and found only limited transfer effects. One could also note that the results from a recent multidomain (updating, shifting, and inhibition) training study conducted with young and old adults showed only near transfer effects (Sandberg et al., 2014). Soveri et al. (2013) studied set shifting training with young adults and found no significant transfer effects. As regards performance on the trained tasks following set shifting training, only Soveri et al. (2013) reported these effects, finding that the training group outperformed the control group on an accuracy measure.

Set shifting represents a rather well-studied construct in cognitive psychology. In set shifting experiments, participants are first asked to perform more simple tasks (= single tasks) with just one instruction in mind (for example, determining if the number in a number-letter pair is even or odd). In addition, the task includes a mixed task block where the participants have to perform different tasks depending on different properties of the stimuli. For example, they may need to determine if the number in a number-letter pair is even or odd when the pair is presented in a certain location, or to determine if the letter in the pair is a vowel or consonant when the pair is presented in another location. Key measures of set shifting ability include switching cost and mixing cost measures both in RTs and accuracy that were defined in the previous paragraph. A switching cost reflects the generally longer RTs and higher error rates to switching trials compared with repetition trials within the mixed block. In turn, the repetition trials of the mixed block tend to elicit slower and more error-prone responses than the single block trials. This effect is coined as the mixing cost and it is thought to reflect increased monitoring demands in the mixed block (Monsell, 2003). All in all, set shifting calls for several executive processes, such as shifting attention between different aspects of the stimulus, shifting between instructions, retrieving instructions from long-term memory and acting upon them, inhibiting the previous instruction or task set, and overall monitoring (Monsell, 2003). There is also a growing number of neuroimaging studies on set shifting (for a review, see e.g., Ruge et al., 2013). These studies have used different procedures that require participants to shift between varying stimulus-response mappings, spatial locations, abstract goals etc. Recent neuroimaging studies employing multiple types of shifts within a paradigm have revealed both domain-independent as well as domain-specific neural correlates of set shifting (Ravizza and Carter, 2008; Chiu and Yantis, 2009; Muhle-Karbe et al., 2014). One further theoretical division in set shifting tasks is the separation into perceptual vs. rule-based switching. Perceptual switching tasks require reorienting of visuospatial attention, that is, “what/where one should address one's attention,” whereas rule-based switching tasks call for changing goal-directed information (rules), that is, “what one should do” (Ravizza and Carter, 2008). There is evidence that these two aspects of switching differ in terms of behavioral effects and neural recruitment, meaning that one cannot draw general conclusions only on the basis of a single type of a set shifting task.

As mentioned above, set shifting ability declines with age, but there are differences as to which type of switching costs are most affected by age (Verhaeghen and Cerella, 2002; Wasylyshyn et al., 2011). Wasylyshyn et al. (2011) investigated in their meta-analysis the relationships between aging and switching and mixing costs (labeled as local vs. global switch cost in their paper). They found that in general, the switching cost does not seem to be affected by age. In other words, selective attention processes needed for the deactivation and activation of cognitive processes in order to perform switches do not seem to be age-sensitive. However, Wasylyshyn et al. (2011) found that the mixing cost that reflects the ability to maintain two task sets was enhanced in older age, and the effect was not explained by general age-related slowing. Wasylyshyn et al. (2011) speculated that the larger mixing cost in late adulthood could be related to impaired working memory, as previous studies have shown a strong relationship between age-related cognitive deficits and working memory processes. In other words, working memory demands might adversely affect set shifting performance in older adults.

The aim of the present study was to investigate transfer effects of set shifting training in older adults, as only one previous set shifting training study reviewed above has included elderly subjects (Karbach and Kray, 2009), and the extent of the generalization effects of set shifting training is controversial. First, we presumed that the training group would outperform the control group on the trained tasks. Second, in the light of previous studies, we expected to find near transfer effects to untrained set shifting tasks. Here, we also wanted to explore if the expected near transfer effects would show differential results regarding perceptual vs. rule-based set shifting. Third, far transfer effects were expected to be less plausible but possible. Measures of inhibition and working memory updating were included as far transfer measures, as these executive functions are related to set shifting (Miyake et al., 2000). Also, Karbach and Kray (2009) found transfer to these domains in their set shifting training study. Cognitive training studies often include measures of fluid intelligence as far transfer measures, as working memory updating and fluid intelligence are strongly correlated (Engle, 2002). In fact, Karbach and Kray (2009) reported that set shifting training generalized to fluid intelligence. Therefore, we also included measures of fluid intelligence among our far transfer measures. In addition, verbal fluency was included as a far transfer measure, as set shifting, working memory updating and response inhibition, in addition to lexical retrieval ability, are important components for optimal performance on verbal fluency tasks (Henry and Crawford, 2004; Flanagan et al., 2014; Shao et al., 2014), and therefore set shifting training might have an effect even on verbal fluency performance. In addition, memory measures were included as transfer measures because in aging research, one has argued for an interplay between executive and memory functions (Bisiacchi et al., 2008). Finally, the visuomotor speed measure was included as a measure of processing speed. In order to explore how long-lasting the possible training-induced effects were, a one-year follow-up was included.

In the present randomized controlled trial, we used a 15-session long adaptive training regime, and included an active control group. We also wanted to look more closely at perceptual vs. rule-based switching (cf. Ravizza and Carter, 2008), as that has not been investigated in previous set shifting training studies. Therefore, the set shifting measures in our pre-post test battery included both a perceptual part, where responses were given according to location of target, and a rule-based part, where responses required the retrieval of appropriate stimulus-response mappings. Untrained tasks tapping set shifting served as near transfer measures. Far transfer tasks included measures of inhibition, working memory updating, fluid intelligence, verbal fluency, episodic memory, and visuomotor speed. We included at least two tests per cognitive domain (apart from visuomotor speed) to ensure that possible transfer effects are not task-specific (see Shipstead et al., 2012).

## Materials and methods

### Participants

Thirty-six healthy Finnish-speaking older adults recruited from various sources (an adult education center, a sports club for seniors, on bulletin boards etc.) volunteered for the experiment. Initial screening of potential participants was conducted over the phone to exclude those with self-reported neurological or psychiatric diseases. Thereafter, a short neuropsychological assessment was conducted, consisting of a semi-structured interview probing the participants' education, occupation, vision, hearing, possible illnesses, traumatic brain injuries, medication, alcohol and/or drug abuse, and possible alcohol intake during the 24-h period preceding the testing, as well as the Finnish version of Consortium to Establish a Registry for Alzheimer's Disease (CERAD; Welsh et al., 1994; Hänninen et al., 2010), the Logical Memory immediate and Logical Memory delayed subtests of Wechsler Memory Scale—Revised (WMS-R; Wechsler, 1996), the Similarities subtest of Wechsler Adult Intelligence Scale—Revised (WAIS-R; Wechsler, 1992), and Memo-Boston Naming Test (Memo-BNT; Karrasch et al., 2010). Before the neuropsychological assessment, the participants were asked to give their written informed consent. After the assessment, they filled in a Finnish translation of the Edinburgh Handedness Inventory (Oldfield, 1971), the Godin Leisure-Time Exercise Questionnaire^1^ (Godin and Shephard, 1997), and Behavior Rating Inventory of Executive Function–Adult Version (BRIEF-A)^2^ (Roth et al., 2005). They also filled in the Beck Depression Inventory-II (BDI-II; Beck et al., 2004) at home before the pretesting in order to rule out major depressive symptoms, as well as the PK-5 Personality test^3^ (Psykologien Kustannus Oy, 2007). Two participants were excluded after the neuropsychological assessment as they performed below cut-off on several memory measures, and one participant dropped out during the training period, bringing the final number of participants to 33 (19 females and 14 males). The study was approved by the Ethics Committee of the Hospital District of Southwest Finland. The follow-up part of the study was approved by the Ethics Committee of the Departments of Psychology and Logopedics at the Åbo Akademi University. The participants did not receive monetary compensation for their participation.

The participants were first matched in pairs and then randomly allotted to the training group (*n* = 17; 10 women/7 men) or to the active control group (*n* = 16; 9 women/7 men). Variables that were taken into account during matching were education, WAIS-R (Wechsler, 1992) Similarities score^4^, age, and gender. The participants were not aware of their group membership. The groups were comparable in terms of years of education *t*_(31)_ = 0.348, *p* = 0.730 (training group *M* = 14.91, *SD* = 3.55, control group *M* = 14.44, SD = 4.27) performance on the WAIS-R Similarities *t*_(31)_ = −0.196, *p* = 0.853 (training group *M* = 29.53, *SD* = 2.40, control group *M* = 29.29, *SD* = 2.21), and age *t*_(31)_ = 0.173, *p* = 0.864 (training group *M* = 68.76, *SD* = 6.68, control group *M* = 68.31, *SD* = 8.28).

### Procedure

**Figure 1:**
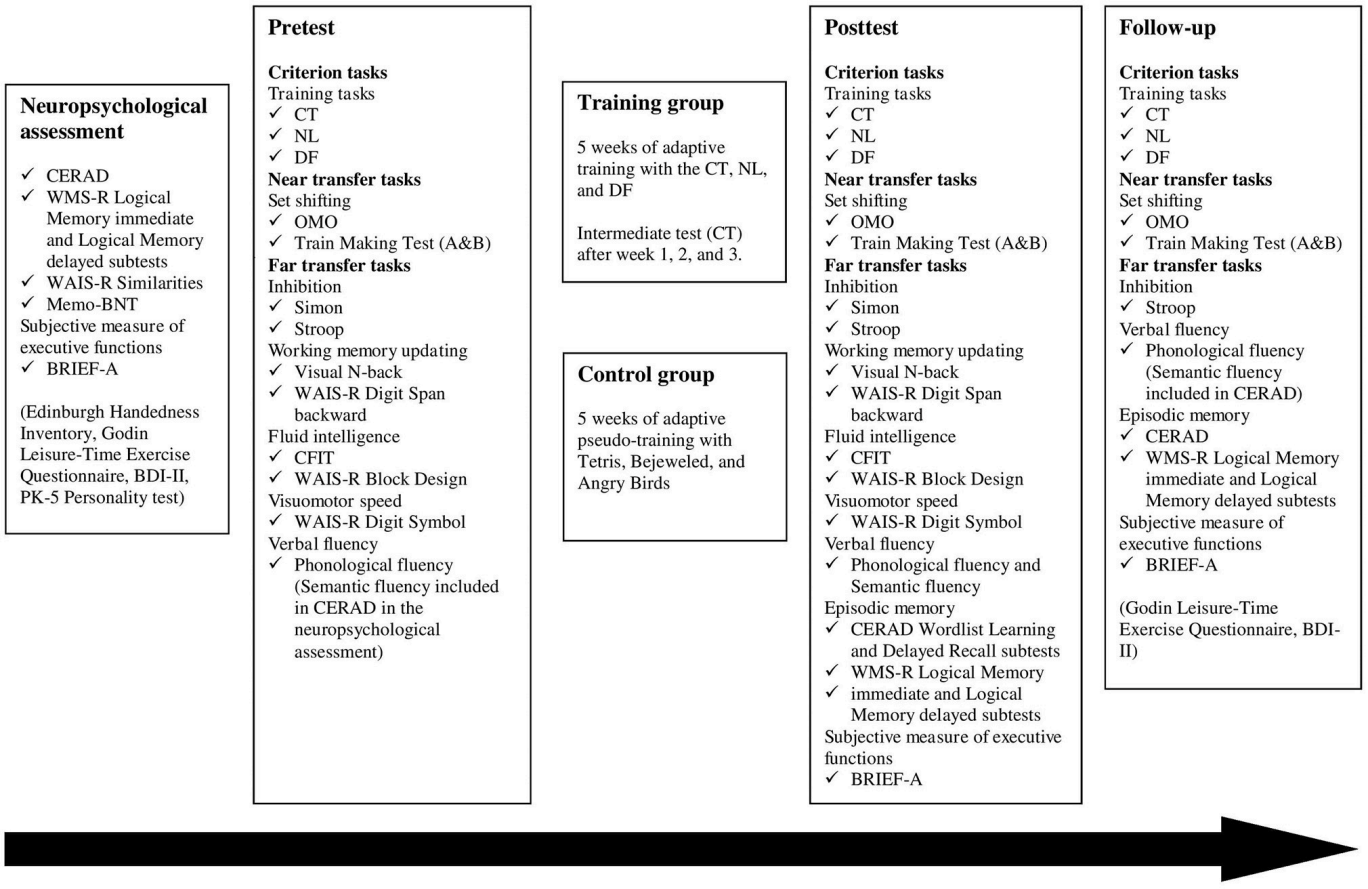
**The experimental procedure including tasks that were administered**. For more detailed information on the procedure and the tasks, see Section Material and Methods.

The experimental procedure including the tasks that were administered is depicted in Figure 1. A randomized controlled trial with a pretest-posttest design was used. Both the training group and the active control group participated in 15 training sessions, 45–60 min/session, three times a week for 5 weeks. The training took place at the university in groups with maximally four people, or individually when needed. All participants underwent the individually administered extensive pre-posttest battery. The posttest was performed maximally 11 days after training, and there was no group difference with regard to the number of days between the last training (or “pseudo-training”) session and posttest *t*_(31)_ = 0.535, *p* = 0.596. The training tasks were adaptive for both the training group and the control group, with the tasks becoming more difficult as the participants advanced. At pretest, at every training session, and at posttest, all participants rated their level of motivation (on a scale 1–5, where 1 = not at all motivated; 5 = very motivated) and fatigue/alertness (on a scale 1–5, where 1 = very tired; 5 = very alert).

### Procedure and training tasks for the training group

Three computerized set shifting training tasks were used: (1) a Categorization Task (CT) that was a modified version of the Wisconsin Card Sorting Test (Berg, 1948; Soveri et al., 2013), (2) a Number-Letter (NL) task (Soveri et al., 2013), adapted from Rogers and Monsell (1995), and (3) a Dot-Figure (DF) task that was a modified non-verbal version of the Number-letter task.

All training tasks included four difficulty levels. To advance to the next difficulty level, the participants had to pass a level test. The level test was a version of the CT that was at the same difficulty level as the previous week's training task, and it was performed after the last training session of the week. The participants who made <20% errors advanced to the next difficulty level. Exceeding this error criterion would have implied staying at the same level for at least 1 week, but all participants advanced after each level test. As there were four difficulty levels, there were three level tests. After the participants had reached the highest difficulty level (level 4), they stayed on that level for the remaining training sessions. The participants were asked to perform as fast and as accurately as possible throughout training. The order of trials in the training tasks and the level tests was randomized.

#### The categorization task (CT) in training

**Figure 2:**
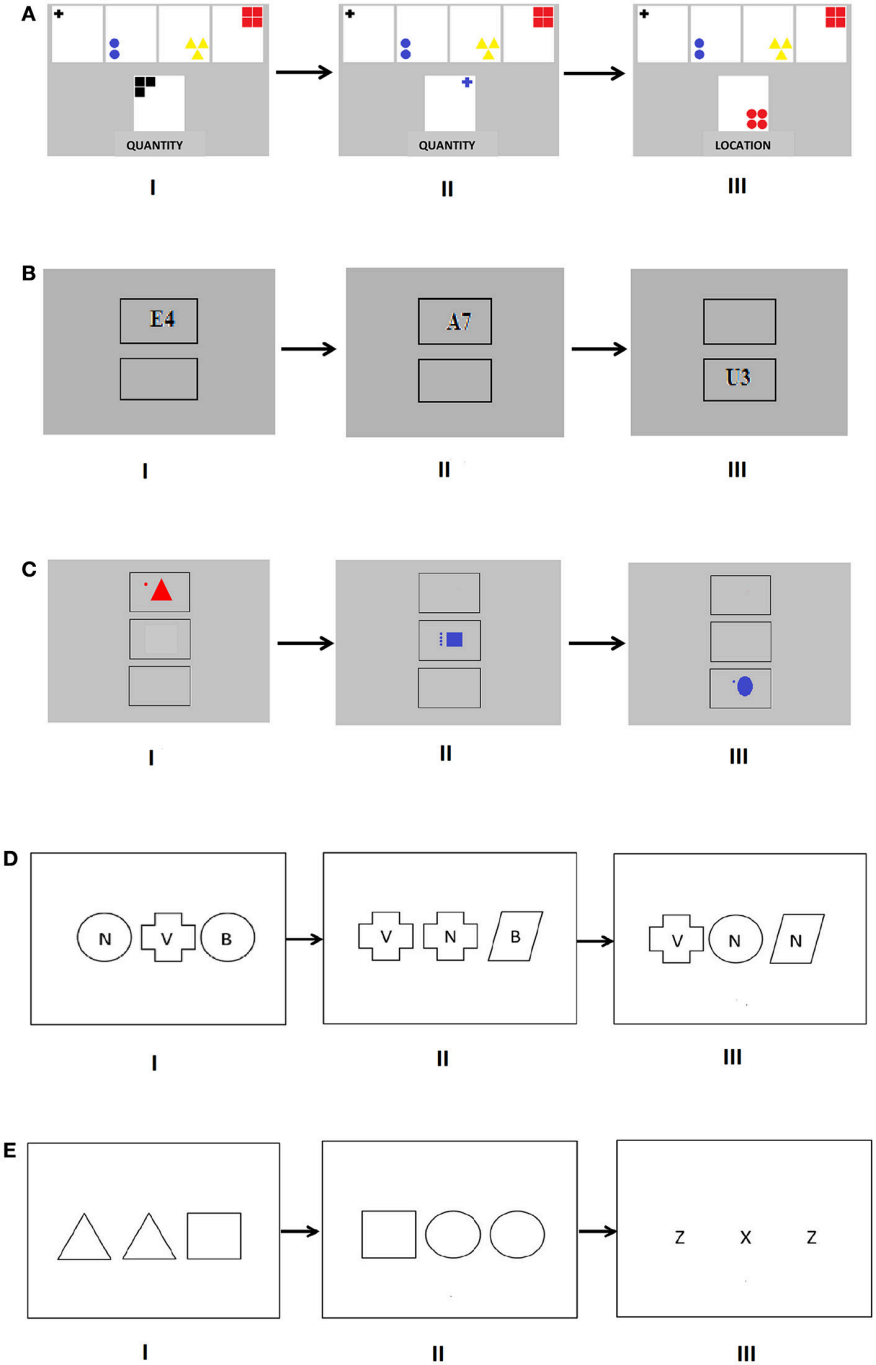
**(A)** *The Categorization Task*. The more difficult version of the task (level 3 and 4). The stimulus cards are at the top of the screen and the response cards appear at the bottom half of the screen, and the written cue is given underneath the response card at each trial. In (I) the given sorting rule is “quantity,” that is, the participant should press “3.” In the repetition trial (II), the participant should press “1,” and in the switching trial (III) the participant is given a new cue “location” and should press “3.” **(B)** *The Number-Letter task*. The easier version of the task (level 1 and 2). Two squares are placed vertically on the screen and the number-letter pair is presented in either one of them. In (I) the participant's task is to decide whether the number is “even” (correct response) or “odd.” In the repetition trial (II) the correct response is “odd,” and in the shifting trial (III) the participant is to decide whether the letter is a “vowel” or a “consonant.” **(C)** *The Dot-Figure task*. The more difficult version of the task (level 3 and 4). Three squares are placed vertically on the screen and the dot-figure pair is presented in one of them. In (I) the participant's task is to decide whether the number of dots is “even” or “odd” (correct). In the switching trial (II) the participant's task is to decide whether the figure is “angular” (correct) or “round,” and in the switching trial (III) the participant is to decide whether the figure is “red” or “blue” (correct). **(D)** *The perceptual part of the OMO task (mixed task)*, where the participant responds by pressing the key that corresponds to the spatial location of the odd stimulus. In trial (I), the correct choice is the middle key that corresponds to the cross. In the repetition trial (II) the correct response is the figure to the right, that is, the parallelogram. In the switching trial (III) the correct choice is the letter “v.” **(E)** *The rule-based part of the OMO task (mixed task)*, where the participant responds by pressing a previously memorized key for that letter or figure (1 = z and triangle, 2 = x and square, and 3 = c and circle). In trial (I), the correct choice is the square. In the repetition trial (I) the correct choice is again the square. In the switching trial (III) the correct response is the letter “x.”

In this task, four stimulus cards appeared in a horizontal line at the top of the computer screen. The task was to match response cards, appearing one at a time, with the stimulus cards, based on different sorting rules that were given. At levels 1 and 2, the four stimulus cards included different *shapes* (cross, circle, triangle, or square), *colors* (red, blue, yellow, or black), and *quantities* (one, two, three, or four figures), and the figures were placed at the center of the cards. The task was to sort the response cards according to these features by deciding which stimulus card had figures of the same shape, color, or number, as the figures on the response cards, based on the sorting rule that was shown underneath each response card. At levels 3 and 4, *location* (upper left, upper right, lower left, or lower right corner) was added as a fourth sorting rule and feature on the stimulus cards. Thus, at levels 3 and 4, the figure was always placed in one of the four corners of the card (Figure 2A). At levels 1 and 3, the sorting rule changed randomly after four to six response cards and at levels 2 and 4 after one to three response cards, regardless of whether the responses were correct or incorrect. Level 1 employed three sorting rules with less frequent shifts (after 4–6 trials with altogether 270 trials), level 2 three sorting rules with more frequent shifts (after 1–3 trials with altogether 270 trials), level 3 four sorting rules with less frequent shifts (after 4–6 trials with altogether 300 trials) and level 4 four sorting rules with more frequent shifts (after 1–3 trials with altogether 288 trials). Task completion took about 15 min. Each difficulty level was preceded by a short practice sequence including all relevant sorting rules. The four response keys, 1, 2, 3, and 4 on the keyboard corresponded spatially to the stimulus cards. The sorting rule was presented for 1,000 ms at the beginning of each trial, and the response card was presented simultaneously until a response was given, or maximally for 3,000 ms. Before moving on to the next response card, audio-visual feedback was given for 1,500 ms. A correct response elicited a high pitch tone and a bright screen, while an incorrect response or no response elicited a low pitch tone and a dark screen. Feedback was given at all difficulty levels. After the task, the number of correct responses, incorrect responses and missed responses were shown on the computer screen. The task included two 1-min pauses, which the participants could end sooner by pressing the Enter key.

#### The number-letter (NL) task in training

At levels 1 and 2 in this task, black number-letter pairs on white background were presented in one of two squares on the computer screen, one square above the other (Figure 2B). When the number-letter pair was presented in the upper square, the participant had to determine if the number was even or odd, and when it was presented in the lower square, the task was to determine if the letter was a vowel or a consonant. Thus, the location of the number-letter pair served as a cue for which task to perform. Number-letter pairs were constructed by combining the vowels A, E, I, U and the consonants G, K, M, R, with the even numbers 2, 4, 6, 8 and the uneven numbers 3, 5, 7, 9. The participants could not anticipate when a number-letter pair shifted from one square to another (switching trial), or when it was shown in the same square as the previous pair (repetition trial, Figure 2B). At levels 3 and 4, a third square was added that was placed underneath the upper two squares, and the number-letter pairs appeared in red or blue. If the pair was presented in the lowest square, the participant had to decide whether the color of the pair was red or blue. Two response keys on the computer keyboard were used, with one response key for vowels, even numbers and red color, and the other for consonants, odd numbers, and blue color. As in the CT, switching trials occurred less frequently at levels 1 and 3 (after 3–5 trials) and more frequently at levels 2 and 4 (after 1–3 trials), thus yielding two squares/less frequent shifts at level 1, two squares/more frequent shifts at level 2, three squares/less frequent shifts at level 3, and three squares/more frequent shifts at level 4. The number of trials at each level was 288, and it took ~15 min to complete the task. Each difficulty level was preceded by a short practice sequence. Every trial began with a blank screen. After 150 ms, a fixation cross appeared in the middle of the screen, being replaced by two or three squares (one of which contained a number-letter pair) after 300 ms. The squares remained on the screen until a response had been given or 3,000 ms had passed. Audiovisual feedback and information about the responses was given in the same manner as in the CT task, and two 1-min pauses were included that could be cut short by pressing Enter.

#### The dot-figure (DF) task in training

This task was identical to the NL task, except that instead of number-letter pairs, dot-figure pairs were used (Figure 2C). At levels 1 and 2, a dot-figure pair presented in the upper square prompted the participant to decide whether the number of dots (that varied between 1 and 4 dots) was even or uneven. When the dot-figure pair was presented in the lower square, the task was to decide whether the figure that was either a triangle, square, circle or oval had an angular or round shape. At levels 3 and 4, a third square was added under the upper two squares (Figure 2C), and at these levels the dot-figure pairs appeared in red or blue. If the pair was presented in the lowest square, the participants had to decide whether the pair was red or blue. Two response keys on the computer keyboard were used: one for even number of dots/angular shape/red color, and the other for uneven number of dots/round shape/blue color. The four difficulty levels followed the same logic as in the NL task.

### Pseudo-training procedure & computer games for the active control group

Three puzzle computer games were used: (1) Tetris (Tetris Worlds, THQ), (2) Bejeweled (Bejeweled 2, PopCap Games), and (3) Angry Birds (version 3.0.0, Rovio Entertainment Ltd). Each game was played for 15 min per session. The games were selected based on their limited demands on set shifting and other executive functions, as well as their appeal to a wide audience. There were 3 difficulty levels in Tetris and Angry Birds. Bejeweled did not have separate difficulty levels, but the game became more difficult due to time pressure, so that the participants had to respond faster as they advanced in the game. Tetris served as a criterion task, in other words, when the participants advanced in Tetris, they could move to the next difficulty level in Angry Birds as well. The participants were asked to perform as fast and as accurately as possible throughout training.

#### Tetris

In Tetris, geometric shapes composed of square blocks each fall down in a matrix, and the participant's task is to move these shapes with the aim to create a horizontal line without gaps. When such a line is created, it disappears and blocks above will fall. When enough lines are cleared, a new level is entered. Difficulty level 1 represented the easiest version of Tetris, and if the participant improved his/her performance in this version during sessions 1–3, the participant moved to the next difficulty level on session 4. Otherwise the participant stayed at the same level until his/her performance improved, whereafter the participant moved to the next level either on session 8 or 12. When the participant improved his/her performance on level 2, the participant moved to the most difficult level either on session 8 or 12, and played at this level for the remaining sessions.

#### Bejeweled

In this game, the participant was to swap one gem with an adjacent gem to form a chain of 3 or more gems either horizontally or vertically. Gems disappeared when chains were formed and gaps were filled by gems falling from the top. Bejeweled was played in a so-called action mode, with the game becoming gradually more difficult due to time pressure.

#### Angry birds

Here the participant used a slingshot to launch birds at pigs in different environments, aiming to destroy all the pigs. As the participant advanced, new sorts of birds became available that had special abilities, which the participant could activate. This game had three difficulty levels. If the participants advanced to the next difficulty level in the criterion task, namely Tetris, they moved to the next difficulty level in Angry Birds as well.

### Pre/post testing

We employed an extensive cognitive test battery including pre/posttest versions of all three training tasks, and tests measuring near and far transfer. Near transfer effects were measured by two set shifting tasks: a modified version of a set shifting test (“odd-man-out” test) previously used by Ravizza and Carter (2008), and the Trail Making Test (A&B; Tombaugh, 2004)^5^. Based on the model of Miyake et al. (2000), tasks measuring inhibition and working memory updating were regarded as far transfer tasks, as were tasks measuring fluid intelligence, verbal fluency and visuomotor speed. Far transfer to inhibition was measured by the Simon task (Simon and Rudell, 1967) and the Stroop task (Lezak et al., 2012). Working memory updating was tapped by the visual n-back task (Cohen et al., 1994) and the WAIS-R (Wechsler, 1992) Digit span subtest (only the backward span is reported here). Fluid intelligence was assessed by the Culture Fair Intelligence Test (CFIT, 1973) and the WAIS-R Block design subtest. Visuomotor speed was measured by the WAIS-R Digit symbol subtest. Furthermore, verbal fluency that taps executive functioning was tested by phonological fluency and semantic fluency tasks. Two episodic memory tests (CERAD wordlist learning/delayed recall and WMS-R Logical Memory immediate/delayed recall) were also performed. Semantic fluency and the memory measures were included in the neuropsychological assessment that was performed already before the pretest session. At posttest, the CERAD wordlist learning and the WMS-R immediate recall were always administered first due to the delayed recall, but the remaining pre/posttests were administered in a random order, both at pre- and post-test^6^. The participants were asked to perform as fast and as accurately as possible when the task at hand required it.

#### Training tasks at pre/posttest

The pre/posttest version of the *Categorization Task (CT)* represented the most difficult level (level 4) of the training task. Four single tasks were always performed first (20 trials each). The sorting rule (shape, color, quantity, or location) was always the same within a single task. The single task was preceded by a practice sequence, in which all the four sorting rules were presented twice, and the practice sequence was presented until the participant made less than 25% errors. After the single tasks, the mixed task block including switching trials was administered, in which the sorting rule changed after 1–3 trials. The number of trials was 144 (72 switching trials, 72 repetition trials). The mixed task block was preceded by a short practice sequence that was repeated once if the participant made more than 20% errors. The order of trials was randomized, but each sorting category and repetitions of the same sorting rule (one, two, or three trials) was presented the same amount of times. Audiovisual feedback was given also in the pre/posttest version of the task. In order to control for situations where the participant might have made a perseveration error that by chance led to the correct answer, the cards were sorted so that the sorting rule could not match with both the previous and the present sorting category. The *switching cost* (the difference between switching trials and repetition trials within the mixed task block) and the *mixing cost* (the difference between repetition trials and single-task trials) in RTs and in the proportions of correct answers were calculated for the CT task.

The *Number-Letter (NL) task* and the *Dot-Figure (DF) task* were administered as follows at pre/posttest. Both tasks started with three single task blocks (32 trials each). In the first single task, number-letter/dot-figure pairs were always shown in the uppermost square (even number/even number of dots or odd number/odd number of dots), in the second single task in the middle square (vowel/angular shape or consonant/round shape), and in the third single task in the lowest square (red or blue color). In all single task blocks, there was an equal number of trials for the two response options. Each single task was preceded by a short practice sequence. If the participant made more than 20% errors, the practice sequence was repeated once. After the single tasks, the mixed task block was performed with 72 switching trials and 72 repetition trials. The order of trials was randomized, and the sequences were balanced for the number of trials per square and for the number of occurrences for each response alternative. The mixed task block was also preceded by a practice sequence that the participant could perform at own pace (max. 10 s per trial). This practice sequence was repeated until the participant made fewer than 20% errors, whereafter a practice sequence with the same ISI as in the actual task was administered once. Audiovisual feedback was given. Similar to the CT, switching cost (the difference between switching trials and repetition trials) and the mixing cost (the difference between repetition trials and single-task trials) in RTs and in the proportions of correct answers were calculated for the NL and DF tasks. All RT measures for the training tasks were based on correct responses only.

To provide more global and possibly more reliable measures of the training tasks, the switching cost, and mixing cost in RTs and in the proportions of correct answers were averaged across the three training tasks. Combining tasks that differ in terms of paradigm and content but nevertheless aim to tap the same domain (here set shifting) has been argued to be a better strategy than combining only homogenous tasks (Schmiedek et al., 2014). The following composits were constructed for the pre/post analyses: composite switching cost in RTs, composite mixing cost in RTs, composite switching cost of proportions of correct answers, and composite mixing cost of proportions of correct answers.

#### Near transfer measures (set shifting)

An “*odd-man-out” (OMO) task* (adapted from Ravizza and Carter, 2008) was used in this study as a near transfer measure of set shifting. The task taps both perceptual and rule-based set shifting, which is of interest here concerning the nature of possible near transfer, as our training tasks required both visuospatial attention and the use of contextual rules. Sets of letters and shapes served as stimuli in the OMO task. The order of trials was randomized. In the *perceptual* part of the task, letters were presented inside figures, three in a row (Figure 2D). The letters used in this task were B, N, and V, and the figures used were a circle, cross, and a parallelogram. The participant was to identify which letter or figure did not match with the other letters or shapes. In a switching trial, the odd stimulus shifted from letter to figure or vice versa. When the odd stimulus was a letter, all the shapes were different and vice versa. Responses in the perceptual task corresponded to the spatial location of the odd stimulus. For example, if the letter or shape in the middle was the odd one, the participant responded by pressing the middle key of the three response keys (1, 2, and 3 on the keyboard). The perceptual task started with two single task blocks (32 trials each). On the first single task block it was always a letter that was the odd one, while on the second single task block it was always a shape. The mixed task block in the perceptual task consisted of 144 trials (72 switching trials, 72 repetition trials). In 72 trials the odd stimulus was a letter while in 72 trials it was a shape. Shifts occurred after one to three trials. Both the single tasks and the mixed task were preceded by a short practice sequence that was repeated once if the participant made more than 20% errors. In the *rule-based* part, the participant's task was to press a key that had previously been memorized for that letter or shape (1 = z and triangle, 2 = x and square, and 3 = c and circle; Figure 2E). In this part of the task, only one feature set was present, i.e., three letters in a row or three figures in a row were shown at a time, and the letters were thus not inside the figure as in the perceptual part of the task. Right before performing the rule-based part of the task, a practice task was performed, requiring the participant to memorize the stimulus-response mappings. In the practice task, the participant received one stimulus at a time, either a letter or a shape, and the task was to respond according to the correct response mapping for that stimulus. First the participant was allowed to perform at own pace (maximum 10 s per stimulus), whereafter the practice task was given with the same ISI as the actual task. This was repeated until the participant made less than 20% errors. The participant received auditory and visual feedback in the practice task: a correct response elicited a high pitch tone and a bright screen, while an incorrect response or no response elicited a low pitch tone and a dark screen. When the participant had memorized the response mappings, the rule-based part with two single task blocks and a mixed task block was performed. Both the single task and the mixed task blocks were preceded by a short practice sequence that was repeated once if the participant made more than 20% errors. In the first single task block, only letters were shown, and the participant had to identify the odd stimulus and respond according to the memorized keys (1 = z, 2 = x, and 3 = c). In the second single task block, only figures were presented, and the response was given according to the memorized keys (1 = triangle, 2 = square, and 3 = circle). In the mixed task block, feature sets (either letters or shapes) alternated (144 trials, of which 72 were switching trials and 72 repetition trials), with shifts occurring after 1–3 trials. All task blocks, both perceptual and rule-based, began with a blank screen. After 150 ms, a fixation cross appeared in the middle of the screen. The fixation cross was replaced by the feature set after 300 ms. The feature set remained on the screen until a response had been given or until 3,000 ms had passed. In the OMO task, the dependent variables were the switching and mixing cost in RTs and the proportion of correct responses in the perceptual and rule-based task, respectively. We also explored possible overall task effects by including the average RTs and accuracy across all task blocks as dependent measures in the analyses. All RT measures were based on correct responses only.

The second set shifting measure was the *Trail Making Test A*&*B* (Tombaugh, 2004). In part A of the test, the participant's task was to draw in ascending order a line as quickly as possible between numbers 1 and 25 that were placed inside circles on a paper sheet. In part B, the circles contained either numbers or letters, and the task was to draw the line alternating between numbers and letters in the sequence, 1-A-2-B etc., as quickly as possible. The processing cost caused by the alternating between numbers and letters, that is, the total completion time of part B (in seconds) minus total completion time of part A, was analyzed.

#### Far transfer measures

##### Inhibition

The computerized Simon task (Simon and Rudell, 1967) and a paper version of the Stroop Test (Lezak et al., 2012) were used as far transfer measures of inhibition. In the Simon task, a red, or a blue square was presented on either side of the computer screen, and the task was to respond according to the color of the square, irrespective of its position that either matched or not with the position of the correct response key. The task was performed by pressing the left key with the left index finger when the square was blue and the right key with the right index finger when the square was red. The task included both congruent (square on the same side as the relevant response key, e.g., red square on the right side) and incongruent trials (square on the opposite side of the relevant response key, e.g., blue square on the right side. Out of the 100 trials, half were congruent and half incongruent. The order of the trials was randomized. The trials were divided into four equally long blocks with a 5-s break in-between. A practice sequence (eight trials) was administered before starting the actual task. A fixation cross was presented at the beginning of each trial. The cross disappeared after 800 ms, replaced by a blank screen for 250 ms. After this, a blue or red square was presented on either the left or the right side of the screen. The stimulus remained on the screen until a response key was pressed or until 1,000 ms had passed. Then the screen went blank for 500 ms before moving on to the next trial. The dependent variables were the Simon effect in RTs and in proportion of correct responses. The Simon effect is the difference between incongruent and congruent trials, and taps the processing cost related to the incompatible location of the stimulus. In the Stroop task, the dependent variable was the Stroop effect, that is, the difference in completion time between naming ink color of conflicting color words (100 trials on a paper sheet) and naming the ink color of sequences of the letter “x” (90 trials on a paper sheet).

##### Working memory updating

Possible transfer effects to working memory updating were measured by the computerized n-back task (Cohen et al., 1994) and the Digit span backward subtest of the WAIS-R (Wechsler, 1992). In the n-back task, numbers from one to nine were presented one at a time at the center of the screen. The task was to remember the previous number (1-back) or the one presented two trials back (2-back). Two response keys were used: the left key for a target, that is, the number was the same as the previous number (1-back) or the one two trials back (2-back), and the right key for a non-target, that is, the number did not match. The total amount of trials was 240 (120 1-back trials, 120 2-back trials). The numbers were divided into 12 blocks of 20 trials each, so that six blocks were 1-back blocks and six were 2-back blocks. The presentation order of the stimuli was pseudorandomized. The 1-back blocks consisted of nine targets and 11 non-targets, and the 2-back block included six targets and 14 non-targets. Before each block, a written prompt informing whether the following block was a 1-back or a 2-back block appeared on the screen together with a picture of a hand indicating the corresponding response keys. After 5,000 ms, the first number was shown, remaining on the screen for 1,500 ms. After this, the number was replaced by a fixation cross for 450 ms. The fixation cross was then followed by the next number. On each trial, the response had to be given within 2,000 ms. The first trial in the 1-back condition and the first two trials in the 2-back condition were excluded from the analysis. The difference in RTs and in the proportion of correct responses between the 2-back and the 1-back conditions were used as the dependent variables for this task. These measures reflect the processing cost caused by the demands on working memory updating in the 2-back condition. In the Digit span backward test, the task was to orally repeat sequences of digits in reversed order. The total score for backward span was analyzed.

##### Fluid intelligence

Fluid intelligence was measured using the Culture Fair Intelligence Test (CFIT, 1973) scales 2 and 4, and the WAIS-R subtest Block design. In CFIT, the participant's task was to find logical relationships between different shapes and figures that were presented on paper. Performance time was limited to 240 s for scale 2 and 180 s for scale 4. Each scale had two equivalent versions, A and B. At pretest, version A of scale 2 and 4 were administered to 17 of the participants (roughly the same number of participants from both groups) and version B to the remaining 16 participants, and vice versa. The dependent variable was the sum of correct responses (scale 2 + scale 4). In the Block design test, the total score of the 9 trials of advancing difficulty (maximum score 51) was analyzed.

##### Verbal fluency

Semantic fluency (producing as many animal names as possible within 60 s) that was included in the neuropsychological screening was performed at posttest as well, and thus also used as a transfer measure. Also phonological fluency (producing words beginning with the phoneme “s” within 60 s) was used as a transfer measure. For both fluency tasks, the number of correct responses was used as the dependent variable.

##### Episodic memory

The CERAD (Welsh et al., 1994; Hänninen et al., 2010) wordlist learning and delayed recall and the WMS-R (Wechsler, 1992) Logical Memory immediate and delayed recall, which were included in the neuropsychological screening, were performed also at posttest and thus used as transfer measures. For the CERAD, we analyzed wordlist learning sum score, delayed recall raw score, and savings score in percent computed by dividing the number of words retrieved on delayed recall by the number of words recalled on the third learning trial (x 100). For the WMS-R Logical Memory, we analyzed immediate and delayed recall.

### Follow-up

The follow-up was conducted 1 year after posttest (plus minus 3 weeks). One participant from the control group declined to participate, and thus 32 out of 33 participants were tested in the follow-up. The follow-up was otherwise similar to the posttest, but the following tests were not included: the Simon task, the visual n-back task, the WAIS-R Digit span, Block design and Digit symbol subtests, the Culture Fair Intelligence Test. The whole CERAD (Welsh et al., 1994; Hänninen et al., 2010) was conducted at follow-up in order to control for possible memory deterioration^7^. Before the follow-up session, the participants were asked to give their written informed consent and after the assessment, they filled in the Godin Leisure-Time Exercise Questionnaire (Godin and Shephard, 1997), and BRIEF-A (Roth et al., 2005), as well as BDI-II (Beck et al., 2004). Motivation and alertness was also surveyed, and some questions concerning the participants' gaming/computer habits were included as well.

### Statistical analyses

ANCOVAs with posttest performance as the dependent variable, pretest performance as the covariate, and group as the between-subjects factor were run on all dependent measures (see Dimitrov and Rumrill, 2003; Senn, 2006). Effect sizes reported as adjusted Cohen's d were calculated using estimated values from the ANCOVA model. For the follow-up, ANCOVAs with follow-up performance as the dependent variable, pretest performance as the covariate, and group as the between-subjects factor were run only on the dependent measures of the training tasks and the OMO task that had been statistically significant or had an *F* > 2 at posttest. Each task was reviewed independently regarding possible exclusion of individual cases. In all tests, the exclusion criteria were being an extreme outlier in accuracy or RTs at pretest or showing evidence of misunderstanding test instructions. Concerning accuracy in the computerized tests, outliers were defined as chance level performance. Regarding RTs in the computerized tests and performance in the paper and pencil tests, outliers were defined as performance laying more than three times the interquartile range above or below the 1st or the 3rd quartile, respectively. There were no outliers regarding RTs.

## Results

### Training results

The means and standard deviations for the composite training scores at pre/posttest and at the follow-up are presented in Table 1, and they are also shown separately for each training task in Table 2 (CT), Table 3 (NL), and Table 4 (DF)^8^.

**Table 1:** **Composite set shifting switching cost and mixing cost scores of the training tasks**.

			**Training (*n* = 17)**		**Control (*n* = 16)^a^**		
		**Session**	**Mean**	***SD***	**Mean**	***SD***	**Sig**.
Reaction times (ms)	Switching cost	Pretest	320.90	119.58	276.11	106.47	N/A
		Posttest	174.46	60.99	287.27	110.99	^*^
		Follow-up	271.60	112.75	287.80	131.31	ns
	Mixing cost	Pretest	400.63	52.51	366.63	72.19	N/A
		Posttest	159.69	53.03	310.81	85.00	^*^
		Follow-up	216.30	81.78	305.33	99.15	^*^
Correct responses (%)	Switching cost	Pretest	−3.94	3.72	−2.36	3.21	N/A
		Postest	−0.69	0.90	−2.15	2.34	ns
		Follow-up	−0.62	1.88	−2.08	2.43	^*^
	Mixing cost	Pretest	−2.33	2.84	−1.50	3.77	N/A
		Posttest	−0.25	1.20	−0.88	1.34	ns
		Follow-up	−0.39	1.17	−1.16	2.43	ns

*Between-group differences at posttest and follow-up were analyzed with ANCOVA*.

a *One participant in the control group declined to participate in the follow-up, leading to a sample size of 15 at that measurement point*.

* *Alpha level is Bonferroni corrected (p = 0.0038 at posttest), ns = not significant*.

**Table 2:** **Performance on the Categorization Task (CT)**.

			**Training (*n* = 17)**		**Control (*n* = 16)^a^**	
		**Session**	**Mean**	***SD***	**Mean**	***SD***
Reaction times (ms)	Switching trials	Pretest	1765.16	288.39	1664.82	247.99
		Posttest	1252.02	188.66	1546.84	243.33
		Follow-up	1433.37	248.93	1578.49	247.33
	Repetition trials	Pretest	1591.12	232.83	1495.24	235.11
		Posttest	1141.50	186.68	1394.55	219.47
		Follow-up	1281.42	190.07	1418.19	222.51
	Single-task trials	Pretest	1301.28	257.79	1214.46	271.76
		Posttest	1007.01	164.18	1156.05	196.77
		Follow-up	1139.02	202.16	1212.46	233.20
	Switching cost	Pretest	174.04	115.84	169.59	88.41
		Posttest	110.52	50.31	152.28	86.73
		Follow-up	151.96	109.07	160.30	112.10
	Mixing cost	Pretest	289.83	128.13	280.78	173.36
		Posttest	134.49	76.55	238.50	146.62
		Follow-up	142.40	121.03	205.74	172.63
Correct responses (%)	Switching trials	Pretest	84.89	10.81	89.67	4.65
		Posttest	96.98	4.77	93.75	4.94
		Follow-up	98.04	2.25	93.61	5.67
	Repetition trials	Pretest	89.54	7.27	93.84	4.78
		Posttest	98.04	3.58	95.49	4.26
		Follow-up	98.12	1.83	93.70	5.55
	Single-task trials	Pretest	91.25	5.10	94.45	4.40
		Posttest	99.04	0.94	97.34	2.70
		Follow-up	98.24	2.21	96.00	4.28
	Switching cost	Pretest	−4.66	7.12	−4.17	4.89
		Posttest	−1.06	1.87	−1.74	2.98
		Follow-up	−0.08	2.43	−0.09	3.26
	Mixing cost	Pretest	−1.71	5.57	−0.62	5.52
		Posttest	−1.00	3.85	−1.86	2.77
		Follow-up	−0.11	3.00	−2.30	5.97

a *One participant in the control group declined to participate in the follow-up, leading to a sample size of 15 at that measurement point*.

**Table 3:** **Performance on the Number-Letter (NL) task**.

			**Training (*n* = 17)**		**Control (*n* = 16)^a^**	
		**Session**	**Mean**	***SD***	**Mean**	***SD***
Reaction times (ms)	Switching trials	Pretest	1499.47	193.32	1368.75	218.12
		Posttest	963.29	140.07	1347.07	159.30
		Follow-up	1262.27	171.81	1364.23	177.00
	Repetition trials	Pretest	1135.99	150.57	1061.81	167.74
		Posttest	751.88	117.28	981.266	150.38
		Follow-up	920.68	139.24	1038.23	149.68
	Single-task trials	Pretest	674.47	90.22	625.53	99.79
		Posttest	590.07	71.92	620.19	81.08
		Follow-up	676.24	99.90	651.21	93.45
	Switching cost	Pretest	363.48	159.29	306.94	185.47
		Posttest	211.41	102.03	365.80	149.52
		Follow-up	341.59	185.86	326.00	154.30
	Mixing cost	Pretest	461.52	100.24	436.28	123.44
		Posttest	161.80	58.71	361.08	115.97
		Follow-up	244.44	121.58	387.03	101.62
Correct responses (%)	Switching trials	Pretest	93.29	7.96	95.33	3.91
		Posttest	99.34	0.72	96.57	4.30
		Follow-up	98.34	1.88	94.65	4.77
	Repetition trials	Pretest	95.83	4.68	97.57	3.27
		Posttest	99.67	0.61	99.13	1.51
		Follow-up	98.86	1.01	97.78	1.73
	Single-task trials	Pretest	98.67	2.07	98.32	1.75
		Posttest	99.75	0.81	99.19	0.92
		Follow-up	99.68	0.51	99.21	1.18
	Switching cost	Pretest	−2.54	6.89	−2.23	2.85
		Posttest	−0.34	0.93	−2.57	3.76
		Follow-up	−0.51	1.86	−3.13	4.30
	Mixing cost	Pretest	−2.84	4.60	−0.75	3.30
		Posttest	−0.07	0.80	−0.06	1.74
		Follow-up	−0.83	1.05	−1.43	1.67

a *One participant in the control group declined to participate in the follow-up, leading to a sample size of 15 at that measurement point*.

**Table 4:** **Performance on the Dot-Figure (DF) task**.

			**Training (*n* = 17)**		**Control (*n* = 16)^a^**	
		**Session**	**Mean**	***SD***	**Mean**	***SD***
Reaction time (ms)	Switching trials	Pretest	1552.03	242.75	1371.69	158.06
		Posttest	971.15	139.48	1288.76	237.39
		Follow-up	1239.90	197.96	1351.75	216.96
	Repetition trials	Pretest	1126.84	172.38	1019.89	109.05
		Posttest	769.69	116.75	945.05	138.55
		Follow-up	918.65	154.10	974.64	114.82
	Single-task trials	Pretest	676.30	114.79	637.04	85.87
		Posttest	586.92	74.11	612.20	68.23
		Follow-up	656.60	107.88	651.40	94.08
	Switching cost	Pretest	425.19	199.40	351.80	137.49
		Posttest	201.46	87.72	343.71	176.24
		Follow-up	321.24	154.10	377.12	183.27
	Mixing cost	Pretest	450.55	112.81	382.85	68.84
		Posttest	182.77	75.76	332.85	99.51
		Follow-up	262.06	101.58	323.24	113.62
Correct responses (%)	Switching trials	Pretest	91.05	7.40	94.63	5.15
		Posttest	99.17	1.00	96.21	3.28
		Follow-up	98.01	3.27	95.49	5.69
	Repetition trials	Pretest	95.67	3.29	95.31	4.67
		Posttest	99.84	0.46	98.35	1.77
		Follow-up	99.26	1.40	98.52	1.53
	Single-task trials	Pretest	98.10	2.45	98.45	1.36
		Posttest	99.49	0.55	99.06	1.03
		Follow-up	99.49	0.67	98.28	1.51
	Switching cost	Pretest	−4.62	5.98	−0.68	6.22
		Posttest	−0.67	1.00	−2.14	2.86
		Follow-up	−1.25	2.98	−3.03	4.53
	Mixing cost	Pretest	−2.43	3.61	−3.14	4.92
		Posttest	0.34	0.83	−0.71	1.83
		Follow-up	−0.23	1.44	0.24	1.86

a *One participant in the control group declined to participate in the follow-up, leading to a sample size of 15 at that measurement point*.

Both the ANCOVA on the composite switching cost in RTs, *F*_(1, 30)_ = 26.671, *p* < 0.001, *d* = −1.84, 95% CI [−2.56, −1.11], as well as on the composite mixing cost in RTs *F*_(1, 30)_ = 59.874, *p* < 0.001, *d* = −2.80, 95% CI [−3.54, −2.06], were statistically significant, due to the smaller switching and mixing costs of the training group at posttest compared with the control group. We also controlled for multiple comparisons with a Bonferroni correction, setting the alpha level to 0.05/4 = 0.0125. Both the switching and mixing cost effects in RTs survived the Bonferroni correction. The corresponding analysis regarding accuracy showed that the composite switching cost was somewhat smaller for the training group *F*_(1, 30)_ = 6.824, *p* = 0.014, *d* = 0.93, 95% CI [0.20, 1.66], but the group effect did not quite reach significance after the Bonferroni correction. The group effect of the composite mixing cost on accuracy did not reach statistical significance, *F*_(1, 30)_ = 2.517, *p* = 0.123, *d* = 0.56, 95% CI [−0.16, 1.27].

### Tasks measuring near transfer (set shifting)

With regard to the near transfer tasks, we corrected for multiple comparisons by setting the alpha level to 0.05/13 = 0.0038

#### The OMO task: perceptual subtest

No significant near transfer effects were seen in the perceptual subtest of the OMO task. Neither the switching cost nor the mixing cost in RTs or accuracy showed significant training-related group differences (*F*s < 1). ANCOVAs were performed also on overall performance both regarding RTs as well as accuracy. The control group performed somewhat faster than the training group in absolute terms, but this was not significant *F*_(1, 30)_ = 2.696, *p* = 0.111, *d* = 0.59, 95% CI [−0.14, 1.33]. The training group performed somewhat better regarding overall accuracy compared with the control group, but this difference did not reach statistical significance, *F*_(1, 30)_ = 3.596, *p* = 0.068, *d* = 0.67, 95% [−0.05, 1.38] (Table 5).

**Table 5:** **Performance on the “odd-man-out” (OMO) task**.

			**Training (*n* = 17)**		**Control (*n* = 16)^a^**		
		**Session**	**Mean**	***SD***	**Mean**	***SD***	**Sig**.
**PERCEPTUAL TASK**							
Reaction times (ms)	Switching trials	Pretest	1954.12	213.14	1819.95	253.20	N/A
		Posttest	1915.15	227.04	1740.81	262.03	N/A
		Follow-up	1997.32	255.51	1787.47	247.05	N/A
	Repetition trials	Pretest	1711.42	272.99	1576.36	199.70	N/A
		Posttest	1688.65	264.87	1498.50	236.60	N/A
		Follow-up	1751.13	312.10	1517.92	287.41	N/A
	Single-task trials	Pretest	1273.27	224.92	1221.41	256.95	N/A
		Posttest	1277.56	233.00	1141.53	213.82	N/A
		Follow-up	1297.83	191.31	1150.99	184.31	N/A
	Switching cost	Pretest	242.70	171.57	243.59	142.76	N/A
		Posttest	226.49	170.00	242.31	112.79	ns
	Mixing cost	Pretest	438.14	192.62	354.95	206.13	N/A
		Posttest	411.10	183.41	356.98	182.71	ns
	Overall RTs	Pretest	1646.27	215.29	1539.24	211.83	N/A
		Posttest	1627.12	221.59	1460.28	219.02	ns
		Follow-up	1682.09	235.29	1485.46	221.04	ns
Correct responses (%)	Switching trials	Pretest	82.52	12.27	85.30	8.23	N/A
		Posttest	88.32	10.48	88.64	8.33	N/A
		Follow-up	84.26	15.90	87.32	12.21	N/A
	Repetition trials	Pretest	85.21	11.48	88.54	7.65	N/A
		Posttest	91.26	6.11	90.71	7.95	N/A
		Follow-up	85.78	14.88	90.56	6.67	N/A
	Single-task trials	Pretest	97.05	3.53	96.47	3.39	N/A
		Posttest	97.72	1.98	96.27	4.15	N/A
		Follow-up	97.91	2.60	96.56	3.39	N/A
	Switching cost	Pretest	−2.69	7.52	−3.24	2.59	N/A
		Posttest	−2.94	6.22	−2.07	2.80	ns
	Mixing cost	Pretest	−11.85	9.95	−7.93	6.28	N/A
		Posttest	−6.46	4.95	−5.56	5.98	ns
	Overall accuracy	Pretest	88.26	8.17	90.10	5.97	N/A
		Posttest	92.43	5.80	91.88	6.40	ns
		Follow-up	89.32	10.42	91.48	6.38	ns
**RULE-BASED TASK**							
Reaction times (ms)	Switching trials	Pretest	1508.15	246.03	1416.79	279.80	N/A
		Posttest	1440.95	224.01	1314.69	177.53	N/A
		Follow-up	1470.90	192.68	1369.72	228.98	N/A
	Repetition trials	Pretest	1422.61	235.08	1309.70	247.84	N/A
		Posttest	1353.17	201.09	1236.49	149.38	N/A
		Follow-up	1402.97	207.29	1296.28	194.20	N/A
	Single-task trials	Pretest	1438.85	238.39	1319.55	186.00	N/A
		Posttest	1375.20	193.77	1249.20	174.01	N/A
		Follow-up	1389.19	191.01	1319.43	199.44	N/A
	Switching cost	Pretest	85.53	54.43	107.08	75.66	N/A
		Posttest	87.78	62.25	78.20	63.96	ns
	Mixing cost	Pretest	−16.23	78.93	−9.84	145.20	N/A
		Posttest	−22.04	75.82	−12.72	109.88	ns
	Overall RTs	Pretest	1456.54	235.59	1348.68	228.79	N/A
		Posttest	1389.77	202.59	1266.79	157.13	ns
		Follow-up	1421.02	191.67	1328.48	201.72	ns
Correct responses (%)	Switching trials	Pretest	92.96	6.32	94.63	6.47	N/A
		Posttest	98.18	1.78	93.84	5.58	N/A
		Follow-up	97.02	2.90	96.34	2.86	N/A
	Repetition trials	Pretest	95.42	3.21	95.40	3.89	N/A
		Posttest	98.04	2.09	94.79	3.88	N/A
		Follow-up	97.39	1.69	96.67	3.31	N/A
	Single-task trials	Pretest	96.87	3.58	95.87	3.81	N/A
		Posttest	99.05	1.52	96.88	3.46	N/A
		Follow-up	98.39	2.13	96.45	3.29	N/A
	Switching cost	Pretest	−2.47	4.91	−0.77	5.22	N/A
		Posttest	0.14	2.41	−0.95	4.01	ns
	Mixing cost	Pretest	−1.44	4.25	0.47	2.74	N/A
		Posttest	−1.01	2.04	−2.08	4.36	ns
	Overall accuracy	Pretest	95.08	3.36	95.30	4.09	N/A
		Posttest	98.42	1.32	95.17	3.67	^*^
		Follow-up	97.60	1.65	96.49	2.50	ns

*Between-group differences at posttest and follow-up were analyzed with ANCOVA*.

a *One participant in the control group declined to participate in the follow-up, leading to a sample size of 15 at that measurement point*.

* *Alpha level is Bonferroni corrected (p = 0.0038 at posttest), ns, not significant*.

#### The OMO task: rule-based subtest

**Figure 3:**
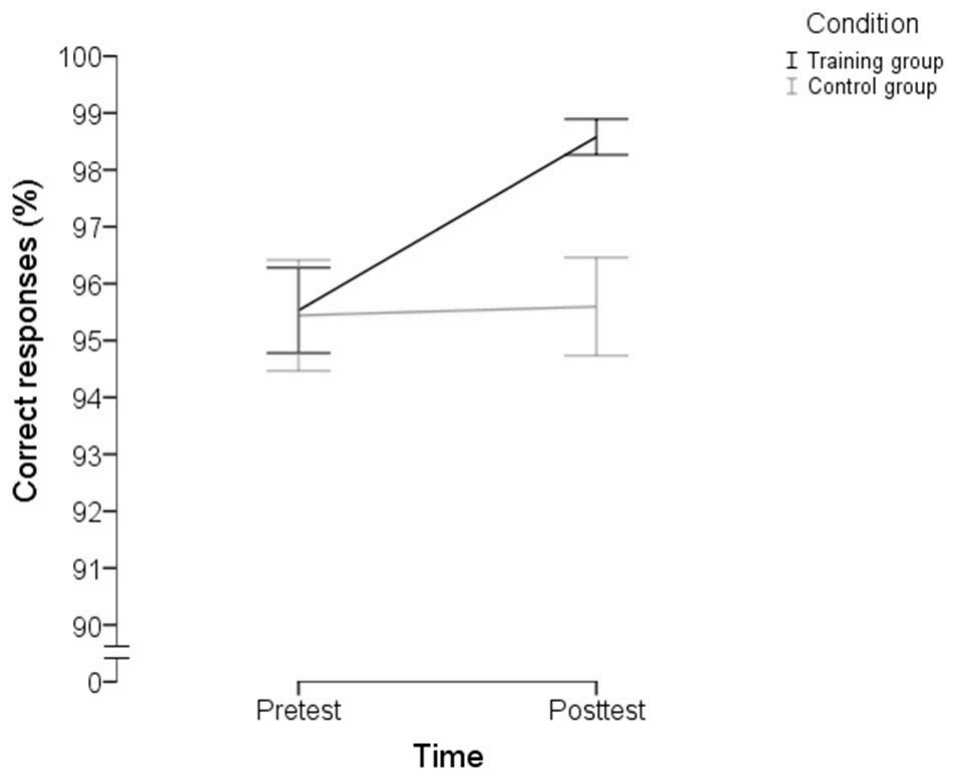
**Number of correct responses (%) across single tasks, switching trials and repetition trials of the rule-based part of the “odd-man-out” near transfer task for the training group and the control group, including standard errors (scale 90–100%)**.

The switching cost and the mixing cost in RTs or accuracy did not differ between groups at posttest as analyzed by ANCOVAs (all *F*s < 2). As above, ANCOVAs were performed also on overall performance, both for RTs and accuracy. No significant group difference in overall RTs was found, *F*_(1, 30)_ = 2.444, *p* = 0.128, *d* = 0.56, 95% CI [−0.17, 1.29]. Regarding overall accuracy, the training group outperformed the control group at posttest, *F*_(1, 30)_ = 14.950, *p* = 0.001, *d* = 1.35, 95% CI [0.64, 2.06] (Table 5; Figure 3), and this finding also survived the Bonferroni correction. In other words, near transfer effects were seen on the rule-based part of the OMO task regarding accuracy.

#### Trail making test A & B

No significant group difference on the switching effect (TMT B minus A) at posttest was seen (*F* < 1; Table 6).

**Table 6:** **Performance on the Trail Making Test A & B**.

		**Training (*n* = 16)**		**Control (*n* = 15)**		
	**Session**	**Mean**	***SD***	**Mean**	***SD***	**Sig**.
A (numbers). duration in seconds	Pretest	44.44	13.86	37.27	14.35	N/A
	Posttest	38.94	11.60	37.07	12.78	N/A
B (numbers+letters). duration in seconds	Pretest	90.81	32.10	86.20	27.31	N/A
	Posttest	80.19	25.03	74.80	22.19	N/A
Switching cost (B − A)	Pretest	46.38	30.97	48.93	20.25	N/A
	Posttest	41.25	19.66	37.73	17.33	ns

*Between-group differences at posttest were analyzed with ANCOVA; ns, not significant*.

### Tasks measuring far transfer

#### Inhibition

No generalization of training gains was observed *n* the *Simon task*, as the main effect of group was non-significant at posttest both with regard to the Simon effect in RTs (*F* < 1) and accuracy (*F* < 2). Nor did the main effect of group on the incongruency effect (word-color conflict completion time minus color completion time) on the *Stroop Test* reach significance, *F*_(1, 29)_ = 2.553, *p* = 0.121, *d* = −0.57, 95% CI [−1.30, 0.16] (Table 7). For *Working memory updating*, no far transfer was not seen, as the n-back effect did not show differentiate the groups on either RTs (both targets and non-targets included), *F*_(1, 28)_ = 2.146, *p* = 0.154, *d* = 0.53, 95% CI [−0.21, 1.28], or accuracy (*F* < 1). The main effect of group on the *WAIS-R Digit span backward* subtest was also non-significant (*F* < 1; Table 7). *Fluid intelligence* tasks showed no significant group difference either on the *Culture Fair Intelligence Test (CFIT; F* < 1) or on the *WAIS-R Block Design* subtest (*F* < 1; Table 8). With regard to *Episodic memory*, the main effect of group on wordlist learning (sum) of the *CERAD* was non-significant, *F*_(1, 30)_ = 2.063, *p* = 0.161, *d* = 0.52, 95% CI [−0.22, 1.26]. Same was true for the main effect of group on the CERAD delayed recall (raw score) and the savings score (both *F*s < 1). The *WMS-R Logical Memory* immediate recall did not show a group difference either (*F* < 1), and nor did the WMS-R logical memory delayed recall, *F*_(1, 30)_ = 2.917, *p* = 0.098, *d* = 0.60, 95% CI [−0.12, 1.31] (Table 8). *Verbal fluency* tasks showed no group differences on semantic fluency (*F* < 1), or on phonological fluency *F*_(1, 30)_ = 2.701, *p* = 0.111, *d* = 0.57, 95% CI [−0.14, 1.28] (Table 8). *Visuomotor speed* was measured with the *WAIS-R Digit Symbol* subtest that did not show any group difference at posttest (*F* < 1; Table 8).

**Table 7:** **Performance on the far transfer tasks measuring inhibition and working memory updating**.

		**Session**	**Mean**	***SD***	**Mean**	***SD***	**Sig**.
**SIMON TASK**							
			**Training (*n* = 17)**		**Control (*n* = 15)**		
Reaction times (ms)	Congruent	Pretest	546.94	76.68	520.02	53.71	N/A
		Posttest	546.69	75.75	508.82	70.26	N/A
	Incongruent	Pretest	586.64	89.61	565.46	68.30	N/A
		Posttest	561.62	81.83	531.71	61.57	N/A
	Simon effect	Pretest	39.70	37.00	45.45	23.61	N/A
		Posttest	14.93	34.33	22.91	39.47	ns
Correct responses (%)	Congruent	Pretest	96.71	3.36	96.93	3.28	N/A
		Posttest	96.94	4.01	96.40	7.10	N/A
	Incongruent	Pretest	95.53	3.84	94.67	4.12	N/A
		Posttest	97.65	2.94	95.33	8.57	N/A
	Simon effect	Pretest	−1.17	4.19	−2.27	4.46	N/A
		Posttest	0.71	3.08	−1.10	3.45	ns
**STROOP TASK**							
			**Training (*n* = 16)**		**Control (*n* = 16)**		
Duration in seconds	Color	Pretest	69.44	9.95	64.38	12.75	N/A
		Posttest	67.81	11.05	63.37	11.42	N/A
	Word-color conflict	Pretest	139.44	23.81	129.63	33.14	N/A
		Posttest	125.63	14.39	124.75	30.12	N/A
	Incongruency cost	Pretest	70.00	18.81	65.25	24.21	N/A
		Posttest	57.81	11.11	61.38	21.44	ns
**N-BACK TASK**							
			**Training (*n* = 15)**		**Control (*n* = 16)**		
Reaction times (ms)	1-back	Pretest	838.86	124.08	757.86	104.40	N/A
		Posttest	762.52	119.15	733.73	111.66	N/A
	2-back	Pretest	1065.36	168.20	951.30	155.55	N/A
		Posttest	979.36	111.17	903.50	113.11	N/A
	N-back effect	Pretest	226.50	114.47	193.44	86.92	N/A
		Posttest	216.84	84.09	169.78	70.52	ns
Correct responses (%)	1-back	Pretest	92.92	4.82	91.34	7.86	N/A
		Posttest	97.19	2.25	95.34	4.20	N/A
	2-back	Pretest	77.53	10.09	74.25	14.81	N/A
		Posttest	82.96	7.31	82.18	10.66	N/A
	N-back effect	Pretest	−15.39	11.38	−17.09	14.70	N/A
		Posttest	−14.47	6.90	−13.16	8.52	ns
**WAIS-R Digit span**							
			**Training (*n* = 17)**		**Control (*n* = 16)**		
Total score	Backward	Pretest	6.06	1.56	6.38	1.31	N/A
		Posttest	6.12	1.69	6.69	1.99	ns

*Between-group differences at posttest were analyzed with ANCOVA; ns, not significant*.

**Table 8:** **Performance on the far transfer tasks measuring fluid intelligence, episodic memory, verbal fluency, and visuomotor speed**.

		**Session**	**Mean**	***SD***	**Mean**	***SD***	**Sig**.
			**Training (*n* = 17)**		**Control (*n* = 16)**		
**CULTURE FAIR INTELLIGENCE TEST**							
Part 2 + Part 4	Total raw score	Pretest	12.71	2.80	12.18	1.94	N/A
		Posttest	12.24	2.86	12.50	2.56	ns
**WAIS-R BLOCK DESIGN**							
	Total raw score	Pretest	26.29	10.62	29.94	7.68	N/A
		Posttest	28.53	9.60	32.25	6.98	ns
**CERAD**							
Wordlist sum score		Pretest	21.12	2.98	23.00	3.52	N/A
		Posttest	23.76	2.68	23.37	4.33	ns
Wordlist delayed recall		Pretest	7.59	1.18	8.13	1.31	N/A
		Posttest	8.06	1.03	8.38	1.45	ns
Wordlist savings score (%)		Pretest	92.35	11.17	97.56	12.06	N/A
		Posttest	94.35	13.31	97.06	15.65	ns
**WMS-R**							
Logical Memory immediate	Total raw score	Pretest	27.35	4.73	26.06	3.57	N/A
		Posttest	30.24	4.93	29.94	3.73	ns
Logical Memory delayed	Total raw score	Pretest	23.53	4.61	23.69	4.38	N/A
		Posttest	28.82	4.52	26.81	4.48	ns
**VERBAL FLUENCY**							
Semantic fluency	60 seconds	Pretest	28.53	5.78	28.56	5.78	N/A
		Posttest	28.53	6.26	27.63	7.26	ns
Phonological fluency	60 seconds	Pretest	17.35	6.30	17.06	6.69	N/A
		Posttest	19.18	7.28	16.25	5.36	ns
**WAIS-R DIGIT SYMBOL**							
	Total raw score	Pretest	45.88	10.48	48.81	14.53	N/A
		Posttest	49.47	10.16	50.44	12.77	ns

*Between-group differences at posttest were analyzed with ANCOVA; ns, not significant*.

### Motivation, alertness and subjective set shifting ability

In order to investigate possible changes in motivation or alertness across the intervention, the relevant survey responses were analyzed with a mixed model ANOVA with motivation/alertness (3 levels: motivation/alertness at pretest, across training sessions^9^, and at posttest) as within-subjects factors and group as a between-subjects factor. A significant main effect of motivation was found *F*_(2, 62)_ = 5.265, *p* = 0.008, as the participants were more motivated at pretest compared with the training sessions/posttest. The motivation x group interaction was non-significant (*F* < 1). The main effect of alertness was not significant (*F* < 2), but the alertness x group interaction was statistically significant *F*_(2, 62)_ = 7.191, *p* = 0.002. Subsequent one-way ANOVAS showed that there were no group differences concerning alertness at pretest or across training sessions (both *F*s < 1), but at posttest a significant group difference was found, *F*_(1, 31)_ = 6.308, *p* = 0.017, with the training group reporting a higher degree of alertness (*M* = 4.32, *SD* = 0.68) compared with the controls (*M* = 3.50, *SD* = 1.15). The set shifting index (raw score) of the BRIEF-A self-report form that was analyzed with an ANCOVA, did not show a statistically significant group difference^10^, *F*_(1, 29)_ = 2.678, *p* = 0.112, *d* = −0.62, 95% CI [−1.39, 0.15].

### Follow-up

#### Training results

The same analyses were run for the follow-up as for the posttest, using pretest as a covariate. The main effect of group on the composite switching cost in RTs did not reach the level of significance at follow-up, *F*_(1, 29)_ = 2.825, *p* = 0.104, *d* = −0.61, 95% CI [−1.36, 0.13], but there was a significant group difference with regard to the composite mixing cost in RTs *F*_(1, 29)_ = 10.900, *p* = 0.003, *d* = −1.21, 95% CI [−1.95, −0.46], due to the smaller mixing cost of the training group at follow-up compared with the control group. Regarding accuracy, a significant group difference for the composite switching cost was seen at follow-up, *F*_(1, 29)_ = 7.292, *p* = 0.011, *d* = 0.99, 95% CI [0.24, 1.73], with the cost being relatively smaller for the training group compared to the control group, but the group effect of the composite mixing cost of accuracy was non-significant (*F* < 2; Table 1). Both statistically significant findings survived Bonferroni correction (0.05/4 = 0.0125).

#### The OMO task

ANCOVAs were run for the overall RTs and overall accuracy at follow-up for both subtests using pretest as a covariate. *Perceptual subtest*. The control group was somewhat faster than the training group at follow-up, *F*_(1, 29)_ = 5.778, *p* = 0.023, *d* = 0.87, 95% CI [0.13, 1.61], but this difference did not survive Bonferroni correction (0.05/4 = 0.0125). The main effect of group regarding overall accuracy did not reach statistical significance (*F* < 1; Table 5). *Rule-based subtest*. No significant group difference on either overall RTs (*F* < 1) or overall accuracy *F*_(1, 29)_ = 2.892, *p* = 0.100, *d* = 0.60, 95% CI [−0.12, 1.33] was found at follow-up (Table 5).

#### Motivation and alertness

At the follow-up, one-way ANOVAS showed no group differences on motivation or alertness ratings (both *Fs* < 1; Table 5).

## Discussion

The present study addressed a potentially important but only scarcely studied area, namely the effects of set shifting training in healthy elderly. In the light of previous training studies, we expected to find improvement on the trained tasks and near transfer effects. Nevertheless, we also wanted to explore whether far transfer effects could be observed. In brief, what we found were strong and long-lasting training effects on the trained tasks, very limited evidence for near transfer, and no far transfer. These results are summarized and discussed in detail below.

Concerning the trained tasks, the training group showed the expected improvement compared to the controls. Our training group outperformed the control group at posttest regarding both switching as well as mixing costs in reaction times. The corresponding posttest effects on accuracy were not statistically significant, although the switching cost accuracy showed a trend for significance in favor of the training group. The analyses on the follow-up performances showed that the training group outperformed the control group on the mixing cost in reaction times and switching cost in accuracy even after 1 year. Thus, the follow-up findings confirmed that the training regime worked, and a 5-week set shifting training can create long-lasting training effects on the practiced tasks. With regard to near transfer, no statistically significant effects were observed on the switching cost or mixing cost in reaction times or accuracy in either part of the odd-man-out task. The switching cost in reaction times in the rule-based part was very small, and the mixing cost was negative. Concerning the overall accuracy and reaction time measures across all task blocks of the odd-man-out task, the rule-based part showed a statistically significant training effect on overall accuracy with a very large effect size (*d* = 1.35). The corresponding overall reaction time measures did not yield a group difference at posttest. Furthermore, no near transfer effects were observed on the Trail Making Test. To sum up, only one measure, overall accuracy on the rule-based part, showed near transfer, indicating a very limited transfer effect. We found no evidence for far transfer on the extensive test battery tapping other executive domains, fluid intelligence, episodic memory, verbal fluency, or visuomotor speed.

Only one of the earlier set shifting training studies (Soveri et al., 2013) has addressed both transfer effects and training effects on the training tasks themselves. Naturally enough, the goal of cognitive training is to obtain improvement on untrained tasks, but verification of training effects on the trained tasks serves as a proof that the training program as such works. In the present study, these effects were verified. In general, improvements on the trained tasks have been the most robust finding in brain training studies (for reviews, see Melby-Lervåg et al., 2016; Simons et al., 2016). This is also true for cognitive training studies that have specifically addressed elderly individuals (Karbach and Verhaeghen, 2014). The fact that these effects were maintained in the follow-up concurs with the largest cognitive training study conducted thus far: the Advanced Cognitive Training for Independent and Vital Elderly study found long-lasting effects on the trained tasks 2 years, 5 years, and even 10 years after training (Ball et al., 2002; Willis et al., 2006; Rebok et al., 2014).

The present results concerning near transfer are broadly in line with most previous set shifting training studies (Minear and Shah, 2008; Karbach and Kray, 2009; Zinke et al., 2012; Pereg et al., 2013) insofar that they have also reported selective near transfer effects. The results also fit well with recent results from an executive process training study including set shifting training that also found limited near transfer effects (Sandberg et al., 2014). A possible reason for the observed near transfer to the rule-based odd-man-out task is that the training tasks may have recruited similar cognitive resources. In general, it has been argued that transfer can take place only if the training and transfer tasks depend upon partly the same cognitive processes and neural systems (e.g., Dahlin et al., 2008a; Waris et al., 2015), and in most executive training studies conducted with older participants that have reported transfer, the transfer has been seen on tasks that are very similar to the trained tasks (Morrison and Chein, 2011; Buitenweg et al., 2012). The Number-Letter and Dot-Figure tasks employed arbitrary cues (placement of number-letter/dot-figure pairs), and the participants had to learn and update the response rules during task performance. Also the Categorization Task and the rule-based odd-man-out task may share some underlying cognitive mechanism(s) as they are both complex in nature, and require several executive processes (Ravizza and Carter, 2008; Naglieri and Otero, 2014). Ravizza and Carter (2008) found that rule-switching in their odd-man-out task that was similar to ours, was related to greater activity in the dorsolateral prefrontal cortex, which in turn has been linked to rule-guided behavior and to context maintenance. Also the Wisconsin Card Sorting Test that the Catergorization Task is based on has been linked to dorsolateral prefrontal cortex activation (Nyhus and Barceló, 2009), and the lateral prefrontal findings in relation to the Wisconsin Card Sorting Test are thought to reflect maintenance of task-set units (e.g., “color”) in working memory (Miller and Cohen, 2001). It might thus be that the near transfer finding in the present study reflects active maintenance of task-relevant information (cf. also Pereg et al., 2013) rather than set shifting. In other words, it is possible that the shared cognitive component between the three training tasks and the rule-based odd-man-out task is working memory updating, an executive process that is required to a higher degree by the rule-based than the perceptual odd-man-out task. This would also be in line with the study by Pereg et al. (2013), as the results from their study suggested that what had been trained as a “set shifting ability” in the study by Karbach and Kray (2009) was not a broad ability, but rather a specific skill related to the unique working memory updating requirements of the training tasks. It is of interest to note that set shifting deficits in late adulthood are usually found when participants have to maintain and coordinate two task sets in working memory (Wasylyshyn et al., 2011). The transfer effect found in the rule-based part of the odd-man-out task was no longer significant at the 1-year follow-up. The fact that no near transfer effects were found on the Trail Making Test may have been due to the fact that this paper-and-pencil test is a rough measure compared with computerized tests that can reveal more subtle performance changes.

The results regarding far transfer effects have been mixed in previous set shifting studies. Minear and Shah (2008) did not include far transfer measures in their study at all, Zinke et al. (2012) found only modest far transfer effects, and Soveri et al. (2013) did not find any far transfer effects. However, Karbach and Kray (2009) found transfer effects to tasks measuring working memory updating, inhibition, and fluid intelligence. Our study differs from the study by Karbach and Kray (2009) in that we used an adaptive training paradigm, and we had a higher number of switches that were distributed somewhat differently in the training. Additionally, Pereg et al. (2013) used the same protocol as Karbach and Kray (2009), but were not able to replicate the far transfer findings of Karbach and Kray (2009). It has recently been argued that especially the far transfer effects seen in executive training studies are not consistent (Shipstead et al., 2010, 2012; Morrison and Chein, 2011; Buitenweg et al., 2012; Melby-Lervåg and Hulme, 2013, 2016), and several previous executive training studies have suffered from methodological shortcomings (e.g., not including an active control group, not using an adaptive training regime, or not including enough transfer measures). We tried to take these criticisms into account by employing an active control group, using an adaptive and long enough training paradigm, and by including at least two transfer tasks per cognitive domain. Still we found only very limited statistically significant near transfer effects.

Some limitations of the present study should be pointed out. First, the sample size was small, which decreases the statistical power and increases the risk for Type II errors. Given the practical challenges of cognitive training studies, Internet-based training experiments that enable larger sample sizes offer one promising way to study further the transfer effects of executive training in the future (e.g., Ngandu et al., 2015). Second, some of the participants performed at ceiling in parts of the training tasks and the odd-man-out task (mainly in the single task blocks), limiting the sensitivity of these tasks in showing possible training-related effects. Third, to rule out possible expectancy effects, future studies should also examine whether the active control group has the same expectations of improvement on the pre/post tasks as the experimental group, as only then can we more confidentially attribute differential improvements to the shifting training (Boot et al., 2013). Fourth, while no motivational differences were found between the groups, the control group reported being less alert than the training group at posttest. However, as the groups were equally alert during pretest and training, alertness was not a confounding factor for the training period. It is unlikely that the higher subjective alertness level of the training group at posttest reflected a general training effect, as in that case one might have expected more widespread transfer. One possibility is that it could be an after-effect of the posttest where the training group performed tasks that were very familiar to them and where they could excel (i.e., the training tasks), whereas the control group had no similar tasks to perform as the computer games were not included in the pre-post test battery. Nevertheless, in absolute terms, both groups displayed adequate levels of subjective alertness at posttest.

In conclusion, we found that set shifting training in the elderly yielded reliable and long-lasting effects on the trained tasks. However, the near transfer effects from this training were very limited.

## Ethics statement

All participants gave their written informed consent to participate in the study. The consent forms were also reviewed by the Ethics Committee of the Hospital District of Southwest Finland, i.e., the subjects were among other things informed that they could quit their participation in the study at any time without a reason, and that the collected data was confidential and kept in a safe place. The follow-up part of the study was approved by the Ethics Committee of the Departments of Psychology and Logopedics at the Åbo Akademi University. No additional considerations, the subjects were healthy.

## Author contributions

All authors listed have made substantial, direct and intellectual contribution to the work, and approved it for publication. More specifically, PG-N designed the study, performed the experiments, analyzed and interpreted the data, and drafted the manuscript. AS, JR, ASN, and ML contributed to the conception of the experiments and they critically reviewed the manuscript. AS and ML also contributed to the interpretation of the data. EE and AN performed the experiments together with PG-N and critically reviewed the manuscript.

### Conflict of interest statement

JR is serving as a neurology consultant for CRST Ltd. None of the aforementioned had any role in the present study, including data collection, preparation or analysis. None of the other authors have any commercial or financial relationships to declare that could be construed as a potential conflict of interest.

## Footnotes

^1^The results of this questionnaire will be reported elsewhere.

^2^BRIEF-A was also filled in at the end of the post testing.

^3^The results of this test will be reported elsewhere.

^4^The WAIS-R Similiarities score was included as a matching criteria, as it is an indicator of general mental ability (Lezak et al., 2012).

^5^A third set shifting measure was also performed, i.e., the CANTAB Attention switching task (AST) (Cambridge Cognition, 2013, www.cambridgecognition.com/academic/cantabsuite/executive-function-tests), but severe problems in the functionality of that test as a set shifting measure led to its exclusion.

^6^However, The CANTAB AST test that had to be discarded was always administered last at pre/posttest due to technical reasons.

^7^Two participants performed below cut-off in the CERAD wordlist delayed recall at follow-up, but as they performed adequately in the other memory measures, they were included the analyses.

^8^The pretest intercorrelations on switching and mixing cost in RTs were low between the CT and NL task, as well as between the CT and DF task across both groups. In turn, the NL and DF task that represent the same task paradigm showed a positive correlation (Pearson's r) regarding both switching cost (*r* = 0.68) and mixing cost (*r* = 0.41).

^9^The motivation/alertness score was missing for one participant in the training group for one training session and the mean was thus calculated based on 14 sessions instead of 15 sessions for that participant.

^10^One participant from the control group was excluded from the analysis due to a highly inconsistent score.
